# X-Entropy: A Parallelized Kernel Density Estimator with Automated Bandwidth Selection to Calculate Entropy

**DOI:** 10.1021/acs.jcim.0c01375

**Published:** 2021-03-13

**Authors:** Johannes Kraml, Florian Hofer, Patrick K. Quoika, Anna S. Kamenik, Klaus R. Liedl

**Affiliations:** † Institute for General, Inorganic and Theoretical Chemistry, Center for Molecular Biosciences Innsbruck (CMBI), University of Innsbruck, A-6020 Innsbruck, Austria

## Abstract

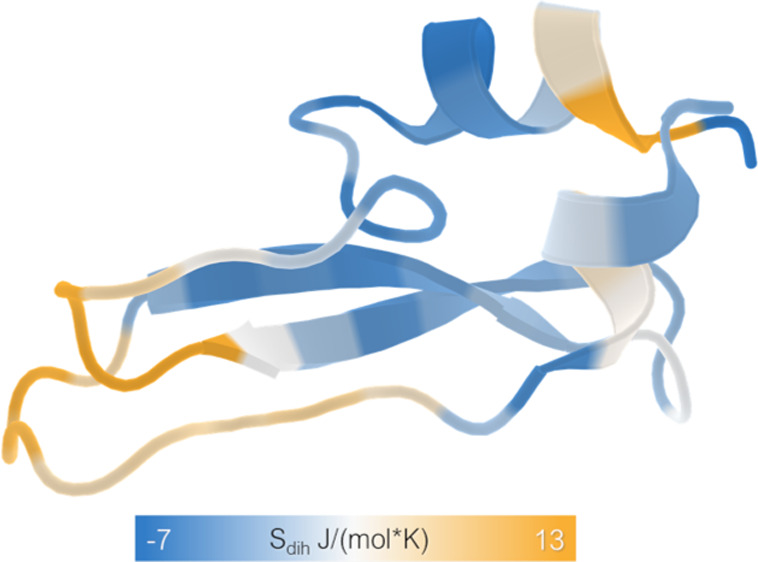

**X-Entropy** is a Python package used to calculate the entropy of a given distribution, in this case, based on the distribution of dihedral angles. The dihedral entropy facilitates an alignment-independent measure of local protein flexibility. The key feature of our approach is a Gaussian kernel density estimation (KDE) using a plug-in bandwidth selection, which is fully implemented in a C++ backend and parallelized with OpenMP. We further provide a Python frontend, with predefined wrapper functions for classical coordinate-based dihedral entropy calculations, using a 1D approximation. This makes the package very straightforward to include in any Python-based analysis workflow. Furthermore, the frontend allows full access to the C++ backend, so that the KDE can be used on any binnable one-dimensional input data. In this application note, we discuss implementation and usage details and illustrate potential applications. In particular, we benchmark the performance of our module in calculating the entropy of samples drawn from a Gaussian distribution and the analytical solution thereof. Further, we analyze the computational performance of this module compared to well-established python libraries that perform KDE analyses. **X-Entropy** is available free of charge on GitHub (https://github.com/liedllab/X-Entropy).

## Introduction

Biomolecules constantly fluctuate between various conformations.^1,2^ Therefore, all physicochemical properties correspond to an ensemble of structures with varying probabilities and not to a single structure alone.^3−5^ It is well established that countless physiological processes, such as biomolecular recognition,^6−8^ catalytic activity,^2^ or drug binding,^9^ are directly linked to a biomolecule’s conformational ensemble. Thus, improving our fundamental understanding of these mechanisms relies on a robust characterization of conformational ensembles. In particular as the relevance of biopharmaceuticals steadily increases, also the thorough exploration of protein dynamics becomes ever more important. Molecular dynamics (MD) simulations are a vital tool to study the conformational flexibility of biomolecules, as they capture conformational ensembles in atomistic detail with reliable state probabilities.^3,9^ One major challenge in working with MD simulations is the intractable complexity of the raw output data. As the motions of biomolecules comprise large domain movements as well as delicate side-chain rearrangements, numerous analysis tools are available to characterize the captured ensembles. Typical analysis approaches range from interaction- or contact-based analyses, like H-bond, ionic, or native contact analyses, over structural characterizations like 1D- and 2D-RMSD, or clustering analyses, to flexibility metrics, like RMSF, or conformational entropy analyses. In this work we focus on a residuewise dihedral entropy metric, previously presented by our group.^10,11^ A major advantage of this metric is that it is completely alignment-independent and quantifies local flexibilities directly from the fundamental thermodynamics encoded in the simulation. The presented approach is based on a plug-in bandwidth selection method presented by Botev et al.,^12^ which facilitates automatized bandwidth optimization. The entropy is then calculated by integrating the probability density functions (PDF) of the individual backbone dihedral angle distributions of the simulated protein. It is worth mentioning that we are calculating the classical coordinate-based dihedral entropy and use a 1D approximation of the entropy. There are other approaches for calculating the dihedral entropy, e.g., quasiharmonic calculation,^13^ 2D Entropy,^14^ MIST,^15^ or the use of Gaussian Mixtures.^16^ In contrast to our approach, these aim at calculating the total entropy of the entire system whereas our approach calculates localized entropies of the individual residues. The sum of these local entropies can be considered an approximation of the total entropy in the system, i.e., the approximation that neglects all higher order terms to the entropy.

In this application note we present a new, complete, and self-contained package, which calculates dihedral entropies on an input data set of dihedral angles. The package consists of a C++ backend, which performs the calculations, and a Python frontend, which serves as API for the user. This setup allows for an easy incorporation of the package in any Python-based analysis workflow. The C++ backend performs initial binning of the input data, followed by the kernel density estimation (KDE) itself. The kernel bandwidth is optimized via plug-in selection, and the resulting PDF is integrated to yield an entropy. To accelerate the calculation, the C++ backend was parallelized using the OpenMP library.^17^ The Python frontend provides the user with prewritten functions for data processing as well as the ability to pass weights for each input frame to the calculation. Consequently, the method can be applied to classical, as well as enhanced (nonequilibrium), simulations. A detailed description of the theory and the implementation can be found in the following section.

While the focus of this application note lies on the calculation of dihedral entropies from biomolecular simulations, the **X-Entropy** package was designed and implemented for general purpose applications. This means that the KDE can be directly accessed from the Python frontend and applied to any binnable, 1D input data.

## Theory and Implementation

The entropy of a continuous system can be calculated via eq 1. The calculation of the integral can easily be performed numerically.^18^
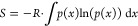
1where *p* is the probability density of the observable *x*, *R* is the gas constant, and *S* is the entropy in this observable. The calculation of the entropy for dihedrals is thus straightforward and can be done numerically, once a continuous PDF has been found. However, the main challenge is to find this underlying PDF. A prominent way to achieve this is to use a KDE and the most common KDE uses a Gaussian function as a kernel. The kernel in a KDE is applied on each data point individually and serves to approximate density away from the exact position of the data points. The Gaussian KDE for any PDF can be written as
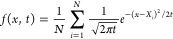
2where *N* is the number of Gaussian kernels used to represent the PDF. In most cases, this is equal to the number of data points. However, it can also be the number of bins in case of binned data, then a factor representing the number of data points within the bin has to be added, more details can be found in the Supporting Information (SI), section 2. *X*_*i*_ is the location of the Gaussian function, and *t* is the squared bandwidth of the Gaussian kernel, i.e., the squared variance of the Gaussian. Finally, *x* is the location where the density is calculated at.

Of all the parameters mentioned above, only the bandwidth (*t*) is unknown. Many implementations use empirical estimators to calculate this bandwidth. This works well for cases, where enough data points are available. We use a plug-in bandwidth selection to calculate the optimal bandwidth, as introduced by Botev et al.,^12^ which allows better performance even with fewer data points. In order to guarantee a rapid calculation of the KDE, we use a fast Fourier transform approach. However, for this approach binned data has to be used. Therefore, the initial data is binned first. The number of bins for this part will be referred to as resolution. Then the bandwidth is calculated using the plug-in bandwidth approach mentioned above, again details can be found in the SI, sections 2 and 3. Finally, we calculate the PDF, using the KDE. This PDF can then be used to calculate the entropy via any numerical integration scheme.

We want to mention here that we are only considering 1D data, also for the entropy calculation of dihedrals. This is a non-negligible approximation, when calculating the total entropy of the system, as the dihedrals in proteins are usually correlated. However, as shown by Polyansky et al.,^19^ the 1D entropy, i.e., not considering cross terms for the entropy, correlates decently well with higher orders, i.e., *R*^2^ = 0.75 for the pure entropy, this improves substantially when calculating entropy differences *R*^2^ = 0.93.

We use a combination of Python, Cython,^20^ and C++ to maximize the usability and the speed of **X-Entropy**. C++ is used in the backend, to guarantee fast and efficient calculations. For the calculation of the fast Fourier transformation, the FFTW library is used.^21^ Due to the widespread use of Python in many analysis pipelines, it is the natural choice for the frontend. Cython is used to connect the backend to the frontend. It is worth noting that some initial analysis is already performed on the Python side.

**Scheme 1:**
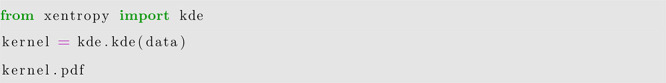
Example Code to Obtain the PDF of a Chosen Observable Stored in the Data Variable

**Scheme 2:**
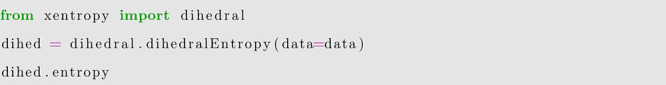
Example Code to Obtain the Entropy of a Set of Dihedrals Stored in the Data Variable

The Python package was developed with an eye on usability and versatility. Despite our focus on dihedral entropy and working with dihedral data, the package was kept as generalized as possible, so that *any* data can be analyzed with the package. Therefore, we allow access to the KDE itself, as well as to our dihedral entropy calculation class. When working with dihedral data, the user can then use this class to analyze the dihedrals that were gathered through third-party software (e.g., cpptraj from AmberTools^22^); compare Schemes 1 and 2.

The interface was designed in a way that entropies of flexible molecules may be calculated in the same scheme as other data distributions. We provide illustrating examples including code and visualization in the version controlled online repository on GitHub: https://github.com/liedllab/X-Entropy.

We implemented a selection of rules of thumb for the estimation of the *optimal* resolution presented in the SI, section 6.1. The resolution can therefore either be chosen by the user, or calculated with one of the different rules of thumb. We apply a specialized resolution selection for dihedral data, as there is more information available for dihedral data, i.e., dihedrals can only have a value between −180° and 180°. Additionally, dihedrals are periodic in higher and lower ranges. Therefore, we use another method, as described in the SI, section 6.2.

Furthermore, the package allows the user to reweigh data from accelerated MD simulations. We use a straightforward reweighing approach as introduced by Miao et al.^23^

## Application and Illustrating Examples

To demonstrate the versatility of our module, we evaluate its accuracy and performance. Therefore, we quantify the deviation of the calculated entropy of samples from a Gaussian distribution with the analytical result. Furthermore, we show a benchmark of the performance of **X-Entropy** against other established python modules. We show that the computational performance of **X-Entropy** is superior to other implementations, especially since we use a plug-in selection approach for automatized bandwidth optimization. Finally, we show exemplary data on how to obtain the conformational entropy of biomolecules from molecular dynamics simulations (MD).

### Analytical Solution for Entropy of Gaussian Distribution

#### Accuracy of Entropy Estimations

As described in the SI, section 5, to analyze the accuracy of the entropy estimation, we compared the estimated entropy from a data set of random numbers with the analytical entropy of the underlying Gaussian distribution. We find that our estimator is able to accurately predict the entropy of the underlying system. For the different data sets, we report a relative error of around 5%, when using a very small sample size (*N* < 10^3^). Once we reach higher sample sizes, we can lower this error to under 1%. Therefore, the accuracy of the prediction is satisfactory, especially for sufficiently large data sets (*N* ≈ 10^3^). Given the asymptotic behavior of the relative errors, which can be seen in Figure S1 in the SI, one can argue that the limiting factor is the size of the data set. In addition, differences from the true value occur, when decreasing the resolution. In summary, there are two factors steering the accuracy of the entropy calculation: on the one hand, the number of observations, i.e., the sample size, and, on the other hand, the resolution, with which the initial binning is performed. Therefore, we suggest using a high resolution when enough data are available but a lower resolution for smaller sample sizes. Thereby, performance may be optimized. Thus, we implemented a procedure to automate the selection of the resolution. This is explained and discussed in the SI, section 6.

#### Performance

To compare the performance of our **X-Entropy** module with other established python packages, we sampled random data sets as mentioned above and evaluated the time it takes on an ordinary PC to perform a KDE of these data. We chose to compare our module with the scipy^24^ and the sklearn packages.^25^

Since these modules are not equally well automatized, we decided to compare them in the following way: Since, sklearn does not estimate the bandwidth on its own, we generally used the estimated bandwidth from **X-Entropy** for the calculation. Furthermore, we evaluated the PDF on the grid, which **X-Entropy** returned for the given resolution. Therefore, in our benchmark, sklearn’s performance actually strongly depends on the calculations of **X-Entropy**, which have been done beforehand. In the case of scipy, we tested two different approaches: First, we used the default way of estimating the bandwidth of the module (which is Scott’s rule^26^), and second, we used the bandwidth estimated from **X-Entropy** for the same data.

**Figure 1:**
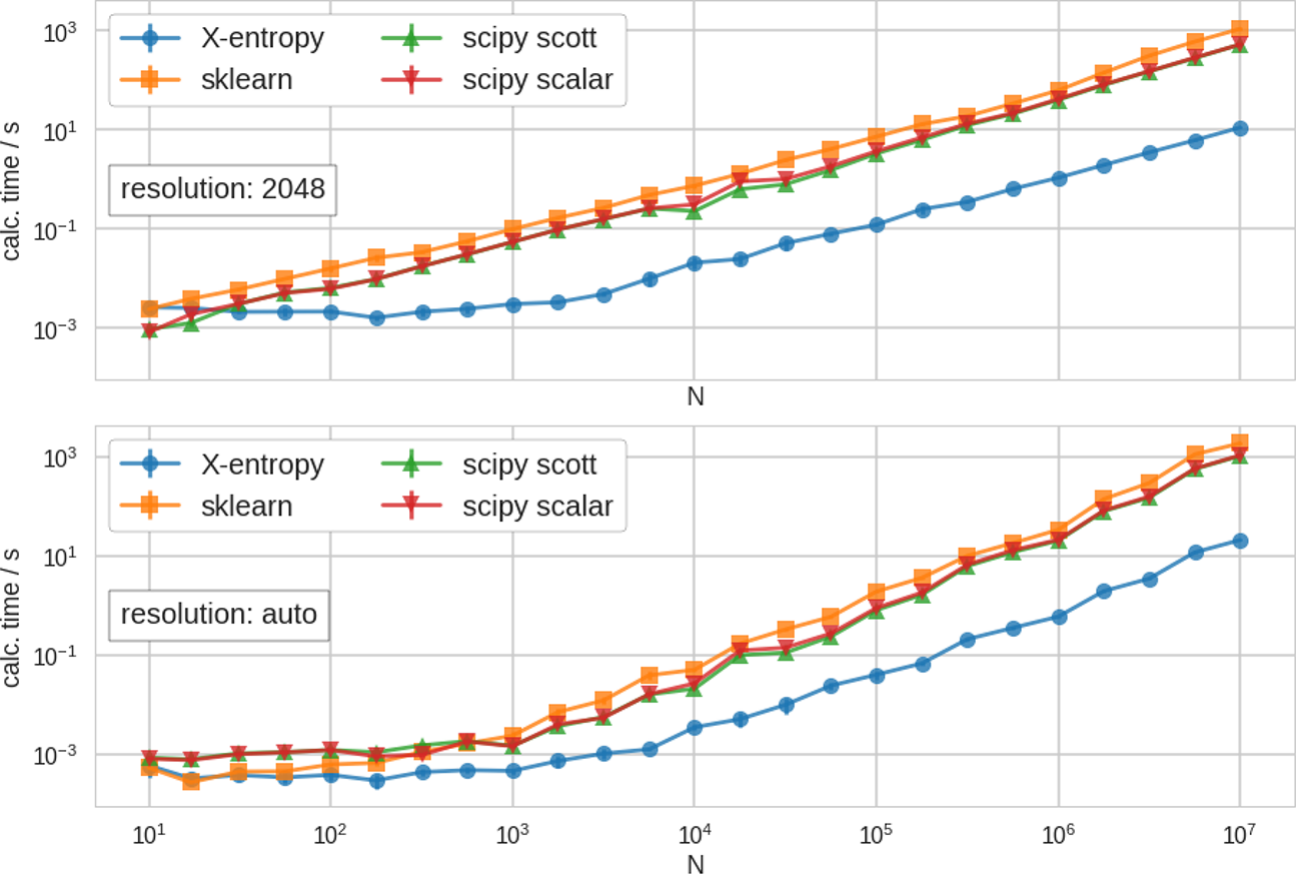
Performance in comparison to other established Python packages. We show the mean calculation time for the KDE of random data sets of increasing size (*N*) with sklearn, scipy, and **X-Entropy**. For scipy, we show two different results: Obtained determining the bandwidth with Scott’s rule (“scipy scott”) and with the bandwidth, which we calculated beforehand with **X-Entropy**. (upper) Results with fixed resolution of *r* = 2048 for all data sizes. (lower) Results with automatically determined resolution. Both panels show the data plotted on a log–log scale.

Generally, the maximum accuracy with which a PDF can be obtained strongly depends on the input samples (see above). Furthermore, depending on the *quality and size* of the sampled data, a better resolution may be obtained. Therefore, we decided to use two different modes for benchmarking: First, we used a high resolution (2048) for all evaluated data sets independent of their size. Second, we used the automated estimation of the resolution, as explained in the SI, section 6. This resulted in an increasing resolution with an increasing number of samples in the data set. As we show in the SI, figure S4, the resulting accuracy of the PDF is still reasonable with both of these approaches. However, the performance obviously differs. In Figure 1, we compare the performance of scipy, sklearn, and **X-Entropy** with the above-mentioned approaches.

### Dihedral Entropy of Alanine Dipeptide

As an illustrative example for the calculation of the dihedral entropy, we performed both, a classical MD simulation (cMD) and an accelerated MD simulation (aMD) of alanine dipeptide. Classical MD simulations are generally hardware limited to the lower microsecond time scale, while most biologically relevant processes occur at much slower time scales.^2^ Numerous enhanced sampling techniques have emerged over the last decades aiming to circumvent this limitation.^27^ Although these techniques vary significantly in their fundamental assumptions, they share the common feature of a bias energy, which is incorporated to accelerate phase space exploration. However, since this bias substantially distorts the free energy surface, reweighting schemes need to be applied to retrieve the system’s unbiased thermodynamics.^23^ Here we use aMD as an example enhanced sampling method to showcase **X-Entropy**’s ability to reweigh biased data on the fly.

Both simulations were performed in a solvated box, at a temperature of 300 K (simulation details can be found in the SI, section 8), and for a total length of 2 μs. We used cpptraj^22^ to obtain the dihedrals of snapshots of these simulations, which we extracted every 10 ps along the trajectory. All data from aMD simulations were reweighed using a McLaurin series of the 10th order.^23^ For convenient usage, we provide the option for **X-Entropy** to calculate a reweighted KDE from the raw data and the weights of the data points. In the SI, Figure S5, we show the distributions of the dihedrals ϕ and ψ with KDE. To reduce the noise caused by the nonoptimal reweighing of the distribution, we use a lower resolution for the processing of the aMD simulation (for visualization in this plot only). The obtained PDF can straightforwardly be transformed to a projection of the free energies on the respective observable. The corresponding 2-dimensional histogram (Ramachandran plot) of these simulations can be found in the SI, Figures S6 and S7.

**Figure 2:**
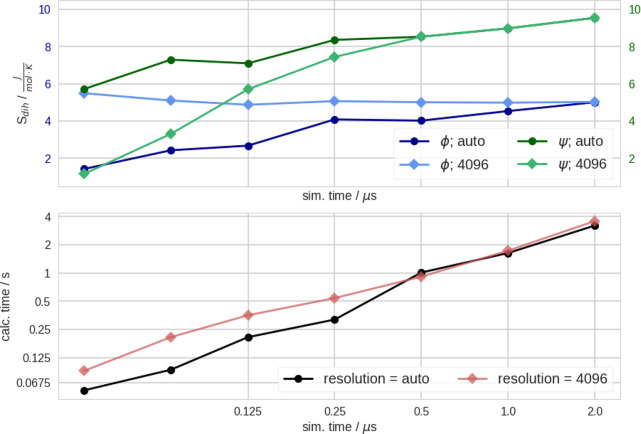
Entropy calculation of the aMD simulation of alanine dipeptide for the dihedrals ϕ and ψ. (upper) Resulting entropies with increasing simulation time: blue ϕ, green ψ. We use dark colors and circles for results obtained with automatic resolution. In light colors and diamonds with fixed resolution, *r* = 4096. (lower) Calculation time for **X-Entropy** with auto resolution with black circles and a fixed resolution, *r* = 4096, in light red diamonds. The resolution calculated with the *auto* keyword can be found in the SI, Figure S9.

Furthermore, we calculated the entropy of both dihedrals with **X-Entropy** for increasing simulation times for both simulations, Figure 2. Since, the spread of the distribution of dihedrals is well-known we apply a modified routine to determine the optimal resolution for KDE of dihedral distributions. For large data sets of dihedrals, the resolution for the KDE is 4096 per default. For a detailed discussion, see the SI, section 8. We report that the processing of the data of this 2 μs aMD simulation took ∼2.6 s altogether (200 000 data points for each dihedral). We obtained dihedral entropies of *S*_ϕ_ = 5.01 J/(mol K) and *S*_ψ_ = 9.54 J/(mol K), respectively. These results are completely in line with the results of the cMD simulation, which can be found in the SI, section 8.3.

### BPTI

**Figure 3:**
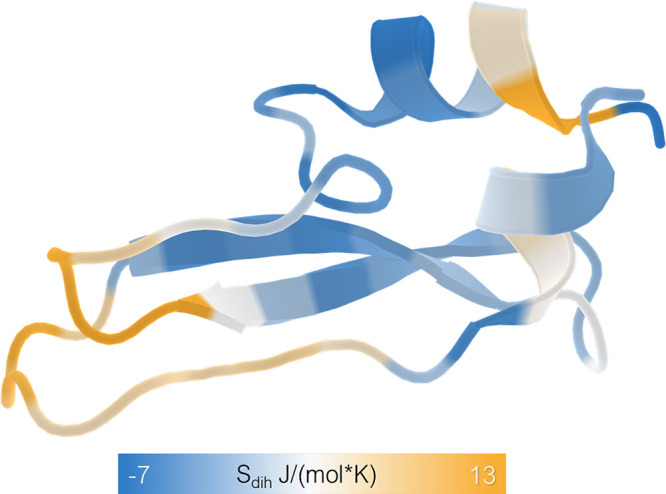
Residuewise dihedral entropy. To illustrate potential applications of **X-Entropy** we show the residuewise dihedral entropies captured in a 1 ms trajectory projected onto the molecular structure of BPTI (PDB 5PTI).

To illustrate the performance on very long simulations, containing large amounts of data we analyzed a BPTI simulation performed in the D.E. Shaw Research lab of just over 1 ms length.^28^ The simulation contains 103 105 frames, with frames stored each 10 ns. As a first step, we calculated the backbone ϕ and ψ dihedral angles from all snapshots of the trajectory (114 in total). These were subsequently analyzed using **X-Entropy**. In Figure 3, we visualize the flexibility of BPTI captured in the full 1 ms trajectory using dihedral entropies. Similar to previous studies^8,11^ the calculated entropy for the backbone dihedrals of each amino acid was mapped onto the crystal structure for an intuitive representation of the protein’s dynamics. We use this as an example for a potential analysis tool-chain starting from simulation data, using Python for the analysis and visualizing the results using PyMol.^29^

In the SI, Figure S10, we further present a comparison of the automated resolution selection and a very high resolution, of 4096. Additionally, we compared the entropy with the RMSD, as another structural analysis method. For the calculation of the entropy, we report a wall clock time of 75.6 s, for all 103 105 frames comprising the BPTI simulation on a standard PC.

## Conclusion

We have shown that our results for the entropy are accurate and robust. In the showcase of Gaussian distributions, we have shown that any deviations from the exact analytical result stem from nonideal sampling or finite resolution. Furthermore, we evaluated **X-Entropy** to be strikingly fast overall. In fact, it outperforms state-of-the art modules, especially for large data sets. Moreover, it can conveniently be used to perform a KDE for arbitrary data without any prior knowledge. First, due to the very straightforward API and second, due to the implemented processing of the data: We automatically provide reasonable estimates of the required resolution and reliably and accurately determine the bandwidth by a plug-in selection method. These automations are of particular convenience, if numerous data sets or simply data sets of high variability are being processed. Most available alternative tools do not provide reasonable estimates for either resolution or bandwidth of the KDE. Choosing these values appropriately by hand can be very tedious. Therefore, we conclude that our tool may be used to accurately, rapidly, and conveniently obtain the PDF of any arbitrary data and calculate the entropy from that.

The conformational entropy of biomolecules is of particular importance for computational chemistry and biophysics. This property may be quantified by the dihedral entropy of these molecules. With **X-Entropy**, we have presented an extremely fast and convenient tool to perform such calculations for arbitrary molecules. Exemplarily, we calculated the classical, coordinate-based dihedral entropy of alanine dipeptide from a simulation of multiple microseconds. This calculation took a few seconds on an ordinary PC and is in good agreement with prior publications.^11^ Furthermore, we used our tool on an extremely long simulation of a relatively small protein. We report not only good performance but also very reasonable correlation of the calculated conformational entropy with other conformational descriptors (RMSD).

These types of calculation are achievable with **X-Entropy** with as much as three lines of code. Hence, it is a particularly useful tool for the calculation of localized, classical conformational entropies of proteins, polymers, or any other molecule.

## Supporting Information Available

Additional technical details on the KDE, the algorithm, and the nummerical integrators we used. Data on the accuracy of the entropy estimation, our choice of resolution, and other KDE modules. Finally, data and simulation details for the two simulated systems, alanine dipeptide and BPTI (PDF)

## Notes

The **X-Entropy** code is available free of charge on GitHub, with example code included https://github.com/liedllab/X-Entropy.
