# Supplementary material for: X-Entropy: A Parallelized Kernel Density Estimator with Automated Bandwidth Selection to Calculate Entropy
Source: J Chem Inf Model. 2021 Mar 13;61(4):1533–8. doi: 10.1021/acs.jcim.0c01375 (PMC8154256; doi:10.1021/acs.jcim.0c01375)
Supplement: Supplementary file 1 — ci0c01375_si_001.pdf [file ci0c01375_si_001.pdf]

Supporting Information
X-Entropy: A Parallelized Kernel Density Estimator with Automated Bandwidth Selection to Calculate Entropy

Johannes Kraml,[†,‡] Florian Hofer,[†,‡] Patrick K. Quoika,[†] Anna S. Kamenik,[†] and Klaus R. Liedl[*,†]

†*Institute for General, Inorganic and Theoretical Chemistry, Center for Molecular Biosciences Innsbruck (CMBI), University of Innsbruck, A-6020 Innsbruck, Austria*

‡*Contributed equally to this work*

E-mail: Klaus.Liedl@uibk.ac.at

1 Other Approaches for Entropy Calculation

We use a parameter free method to calculate the entropy. We first perform a KDE, to estimate the underlying PDF from the data. However, using a KDE and integrating the underlying PDF is not the only possible way to calculate the dihedral entropy. Gyimesi et al.[1] used a Gaussian mixtures approach, in which they use a variable number of Gaussian functions to estimate the underlying PDF. Their main problem is to analyze the data to find optimal positions for their Gaussians and then estimate the optimal width of the Gaussian function. Compared to this, our KDE approach is more straight forward, as a KDE places a kernel on each data point and only the bandwidth, i.e., the width of the Gaussian function, needs to be optimized. However, the benefit of their method is that they can calculate the differences in entropy in higher dimensions.

Wang and Brüschweiler[2] use another approach, called 2D Entropy, where they calculate the entropy of a set of dihedrals by first calculating a PCA and then estimating the entropy along each mode of the PCA. The term 2D comes into effect, as they represent the dihedral angles as points on the unit circle and have therefore a complex term.

2 Kernel Density Estimation Theory

Any PDF can be approximated using a KDE. Most KDEs use a Gaussian function as their kernel, which in general has the form shown in equation 1.

$$f(x,t) = \frac{1}{N}\sum_{i=1}^{N}\frac{1}{\sqrt{2\pi t}}e^{-\frac{(x-X_i)^2}{2t}} \tag{1}$$

where N denotes the number of Gaussian functions used to construct the KDE, X_i is the location of the Gaussian function. t is the squared bandwidth of the Gaussian Kernel, i.e., the squared variance of the kernel. For binned data, the number of data points in a given

bin have to be considered. This leads to equation 2.

$$f(x,t) = \frac{1}{N_d}\sum_{i=1}^{N_b}\frac{n_i}{\sqrt{2\pi t}}e^{-\frac{(x-X_i)^2}{2t}} \tag{2}$$

where N_d is the total number of data points, N_b is the total number of bins, n_i is the number of data points in bin i, X_i is the location of the Gaussian on bin i, i.e., the center of the bin, x is the location where the PDF should be calculated, t is the bandwidth, as mentioned above. The main problem for any KDE is the estimation of the optimal parameter t. The optimal parameter is found by minimizing the error between the estimate calculated via the KDE and the real PDF. Usually, the mean integrated square error (MISE), equation 3, is used to define the error between these two.

$$\text{MISE}\,(t) = E_f \int \left(\hat{f}(x;t) - f(x)\right)^2 dx \tag{3}$$

where $\hat{f}(x;t)$ is the estimation function of the PDF with respect to the squared bandwidth t, whereas $f(x)$ is the true PDF and E_f is the expected value of the sample. Therefore, the MISE depends in a non-trivial manner on the bandwidth, as well as the true PDF. To circumvent these problems, usually the asymptotic estimator of the mean integrated square error (AMISE), equation 4, is used. Thus, the optimal bandwidth for any KDE is the minimizer of the AMISE, i.e., the value that yields the smallest mean integrated square error in the asymptotic case using the given KDE.

$$\text{AMISE}\left\{\hat{f}\right\}(t) = \frac{1}{4}t^2||f''||^2 + \frac{1}{2N\sqrt{\pi t}} \tag{4}$$

This directly leads to the motivation of the bandwidth calculation presented by Botev et al..[3] The optimal bandwidth of a Gaussian function can then be calculated by optimizing the

AMISE, i.e., calculating the minimum of the AMISE (equation 5).

$$_{*}t = \left(\frac{1}{2N\sqrt{\pi}||f''||^2}\right)^{\frac{2}{5}} \tag{5}$$

However, one can see that the optimal parameter for t (denoted as $_{*}t$) depends on the second derivative of f, i.e., f''. The function f'' depends on another optimal bandwidth parameter ($_{*}t_2$). This parameter again depends on the third derivative of f ($f^{(3)}$). In general, $_{*}t_j$ depends on $f^{(j+1)}$, i.e., the j+1'th derivative of f, as can be seen from the more general equation 6.

$$_{*}t_j = \left(\frac{1 + 1/2^{j+1/2}}{3} \frac{1 \cdot 3 \cdot 5 \cdot \cdots \cdot (2j-1)}{N\sqrt{\pi/2}||f^{(j+1)}||^2}\right)^{\frac{2}{3+2j}} \tag{6}$$

Therefore, Botev et al.[3] suggest to use a plug-in bandwidth estimator, which starts by estimating $f^{(5)}$ from the original data and uses the optimized value for $_{*}t_4$ to calculate $f^{(4)}$. This procedure is performed for each $f^{(j)}$ to calculate $_{*}t_{j-1}$ until $_{*}t$ is reached. The fifth derivative was found to be optimal by Botev et al., as higher derivatives do not yield a significant increase in accuracy. Using a Fourier Transformation has two major benefits. First, the calculation of the PDF is faster. Second, calculating the derivatives of the Gaussian kernel is much more efficient in Fourier space. Therefore, we use the Fourier transformation to speed up these calculations, which is especially necessary, as the derivatives have to be calculated very often.

The initial estimation of $f^{(5)}$ directly from the data is the only weakness, as the assumption that this represents the correct fifth derivative of f does not hold. Therefore, $_{*}t$ is calculated from the original data and then used as input for the next iteration, i.e., starting by calculating $f^{(5)}$ using the $_{*}t$ and then calculating a new optimal bandwidth. This cycle is performed until the error between the input bandwidth and the optimized bandwidth is within the error of the double data type. This shows another reason, why calculation in Fourier space is highly beneficial, as the derivatives have to be calculated in every iteration of the process.

3 Algorithm

The algorithm presented by Botev et al. and implemented in the presented python package, works as follows: Initially, the user supplies some data, this can be dihedral data through the dihedral entropy interface, or any other binnable data. The user can additionally choose, whether the resolution, i.e., the number of bins, is chosen automatically. However, to give the user full control, the resolution can also be set by the user. Afterwards, the algorithm performs the following steps:

1. Bin the initial data according to the specified resolution
2. The binned data is then transformed into Fourier space
3. Set $_{*}t$ to the maximum number stored in double precision, set t_b to 0
4. As long as $|_{*}t$ - $t_b| > \epsilon$ perform the following steps, where ϵ is double accuracy.
   - Estimate initial $f^{(5)}$
   - For j = 4; j > 2
     - calculate $f^{(j)}$ using $f^{(j+1)}$
   - Set t_b to $_{*}t$
   - Calculate $_{*}t$ from $||f''||^2$
5. Yield the optimal bandwidth and the optimal density estimate using that bandwidth.

4 Integrators

Within the module, we implemented two different numerical integrators: (1) The first relying on the Riemann integration scheme and (2) the second relying on the Simpson integration for a range of values. Both of these integrators are implemented using OpenMP and parallelized over the range of data.

The Simpson integrator implements equation 7. We use two loops to accumulate the data and sum it up in the same variable, using OpenMP's reduction clause. The variable of the loop is actually incremented by two, saving one multiplication in the process.

$$\int_a^b f(x)dx \approx \frac{\Delta x}{3}\left[f(a) + 2\sum_{j=1}^{\frac{n}{2}-1} f(x_{2\cdot j}) + 4\sum_{j=1}^{\frac{n}{2}} f(x_{2\cdot j-1}) + f(b)\right] \tag{7}$$

The Riemann integrator implements the more straight forward equation 8.

$$\int_a^b f(x)dx \approx \sum_{j=0}^{n} f(x_j)\cdot \Delta x \tag{8}$$

where $x_j = a + j \cdot h$ and $\Delta x = \frac{b-a}{n}$. Note that $x_0 = a$ and $x_n = b$.

5 Accuracy of Entropy Estimation

In order to quantify the accuracy of **X-Entropy**, we compared the calculated entropy of samples from a Gaussian distribution with the analytical solution. Therefore, we used numpy [4] to generate random samples with increasing sample size. In detail, we increased the number of samples per data set from $10^{1.5}$ up to 10^7. In order to judge the uncertainty of the resulting entropy for a given sample size, we calculated the mean result of 25 different data sets per size. For comparison, we calculated the analytical result as

$$S_{gauss} = -\frac{1}{2} \cdot \ln\left(2\pi e \sigma^2\right) . \quad (9)$$

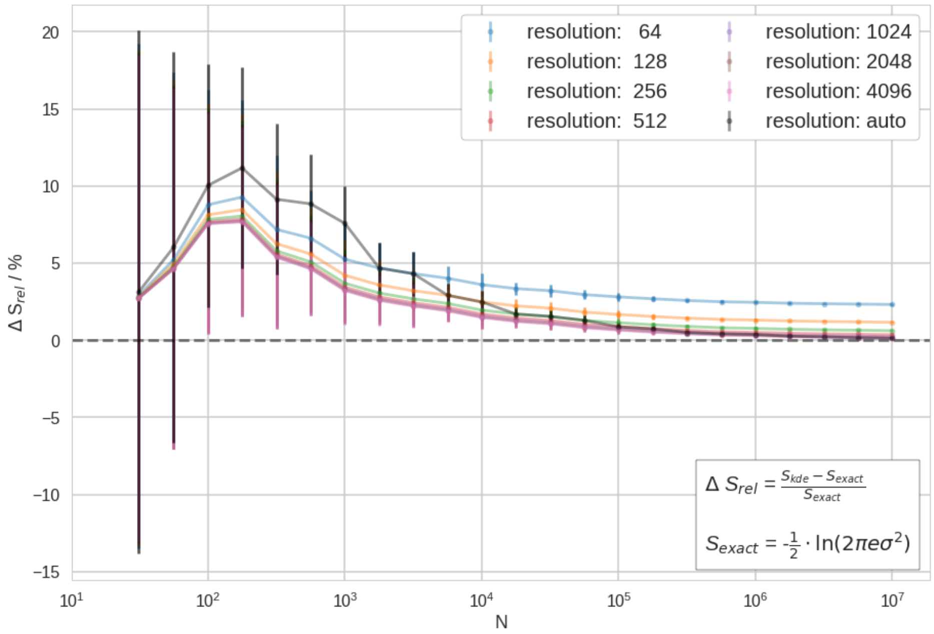

Figure S1: Relative deviations of calculated entropy from ideal result (in %). Entropies have been calculated from KDE with **X-Entropy** of random data sets of increasing size (N). We calculated mean relative differences (ΔS_{rel}), as displayed in the figure. In different colors we show results obtained with different resolutions for the KDE. In black we show the results obtained with the automatic determination of the optimal resolution, eqn. 15.

In figure S1 we show the relative deviations of our entropy estimations from the ideal result of a Gaussian distributions (ΔS_{rel}). There, we plot results obtained with different resolutions in different colors. We used a constant resolution for any size of the data set,

except for the black curve: These results have been obtained with an automatic determination of the optimal resolution, given a certain data set (see below). Generally, we observe an asymptotic progression of all curves for large data sets: The higher the number of samples, the smaller is ΔS_{rel} on average. Conclusively, the maximum possible accuracy is reached in the limit of infinite number of samples. Furthermore, we note that the deviation from the ideal result vanishes with increasing resolution. Obviously, the higher the resolution, the better is the maximum accuracy.

6 Choice Of Resolution for KDE

We implemented two options for the user to choose the resolution: (1) They may give a scalar. (2) They may select from a number of possible rules of thumb to determine the resolution. Generally, the resolution will internally be rounded up to the next power of two (if it is no power of two already). This is being done, because data sizes of powers of two are needed for the fftw. We implemented a selection of rules of thumb, which we list below. Most of these are readily implemented in numpy.[4] Wherever it was possible, we used the respective numpy function.

Besides, we implemented our own routines to automatically determine the *optimal resolution*. Therefore, we distinguish between arbitrary data or dihedral data, specifically. These routines are outlined and explained below.

Conventional Rules Of Thumb

Below we list a number of conventional rules of thumb to estimate the optimal resolution. We chose to order them in increasing complexity. For further reading we recommend.[5] Consistently, we use N to denote the sample size below. Some formulas below, estimate the number of bins (n_h), while others actually estimate the binwidth (h). These quantities are mathematically convertible by multiplication/division with the data range. Other quantities, which are needed for the calculation, are defined in place.

Square Root Choice (src)

$$n_h = \sqrt{N} \tag{10}$$

Sturges

$$n_h = \log_2 N + 1 \tag{11}$$

Scott's rule

$$h = \sigma \sqrt[3]{\frac{24 \cdot \sqrt{\pi}}{N}} \simeq \frac{3.49 \cdot \sigma}{\sqrt[3]{N}} \tag{12}$$

σ is the standard deviation of the input data.

Freedman-Diaconis Estimator[6]

$$h = 2\frac{IQR}{\sqrt[3]{N}} \tag{13}$$

IQR denotes the interquartile range of the data.

Doane's Formula

$$\begin{aligned} n_h &= 1 + \log_2(N) + \log_2\left(1 + \frac{|g_1|}{\sigma_{g_1}}\right) \qquad (14) \\ g_1 &= \left\langle \left(\frac{x-\mu}{\sigma}\right)^3 \right\rangle \\ \sigma_{g_1} &= \sqrt{\frac{6(N-2)}{(N+1)(N+3)}} \end{aligned}$$

where g is the skewness of the data. Furthermore, $\langle\ \rangle$ denotes the calculation of the mean; x is the mean and μ is the median of the input data; σ is the standard deviation

6.1 For Arbitrary Data

For pragmatic reasons, we included an additional rule of thumb, which, to our knowledge, has not been implemented anywhere before. It is a slight adaption of the *square-root choice*. Therefore, it is similar to the automatic determination of the number of bins of numpys ***histogram_bin_edges*** function. The functions listed above are actually meant to determine to optimal number of bins in a histogram, which is clearly not exactly the same as determining the optimal resolution for a KDE. Due to the nature of its calculation, a KDE is able to provide some sort of interpolation between histogram bins (depending on the used kernel). Therefore, we decided to choose a rather high resolution in comparison to the conventionally chosen number of bins in a histogram. We experimented with different resolutions and sizes of data sets and came up with the following calculation of the *optimal resolution* (r).

$$r = max\left(32, \sqrt{N}\right), \tag{15}$$

where N is the size of the input data. In figure S2, we visualize r for different methods with increasing data size, including our rule of thumb (labeled with *auto*). This method has been proven to be very robust, with data from arbitrary distributions for very wide ranges of data size.

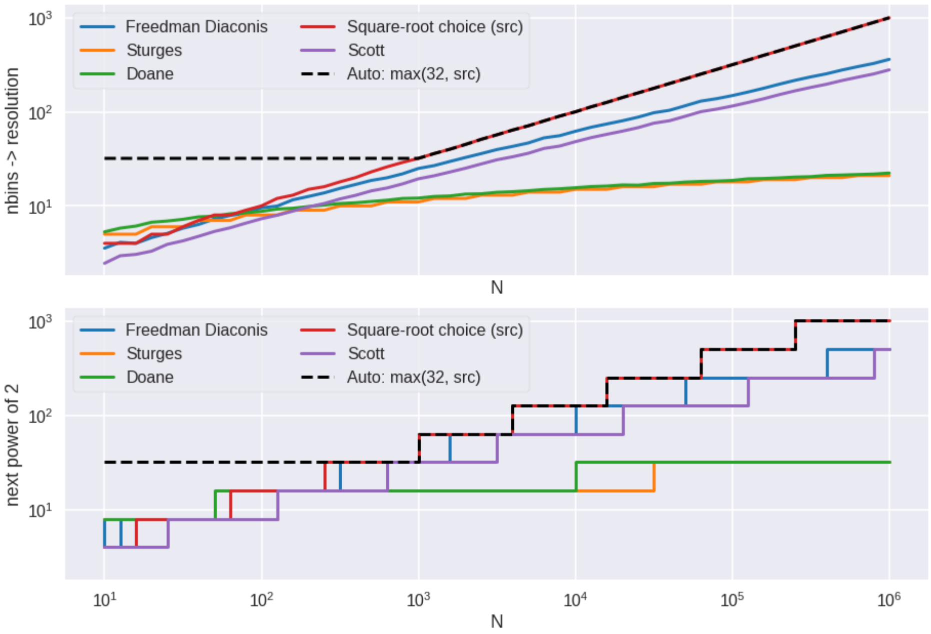

Figure S2: Mean resolutions obtained with different estimators, for random samples from a Gaussian distribution with increasing sample size(N). In the upper panel we show the estimated resolution with different rules of thumb, in the lower panel we show the next power of two, which results from the calculated number of bins of these methods above.

6.2 For Dihedral Data

Considering the additional prior knowledge, i.e., values for the dihedrals range from -180 to 180, or from $-\pi$ to π, and they are periodic in that range, meaning that -180 is equal to 180, we use a more specific approach for the determination of the resolution of the KDE of dihedral distributions. Since, we assume the data acquisition to underlie a certain maximum precision, we pragmatically chose a maximum resolution for the calculation of the PDF of dihedral distributions. This high resolution is only applied automatically for data sets of a certain minimum size. Furthermore, even for very small data sets, we did not want to undercut a certain resolution. The reasons for that are firstly, that dihedral distributions are potentially *multimodal*. Secondly, they spread between $+\pi$ and -π and quite impractically, peaks may be cut off periodically. Therefore, they are *rather widespread* (in comparison to the naive standard deviation of the data set). Hence, we introduce periodic copies of the dihedral data, which is important to keep in mind for the determination of the resolution. Therefore, the specified resolution for the KDE (which needs to be a power of two), is effectively divided by three. We came up with the following empirical formula to automatically calculate the optimal resolution (r) for the pdf of dihedral data:

$$r = min\left\{4096,\ \left[floor\left(\sqrt{N \cdot 6},\ 64\right) + 1\right] \cdot 256\right\}, \tag{16}$$

where $floor$ denotes a floor division and N is the number of samples in the raw data set (*not* the data set with periodic copies). Same as for arbitrary (non-dihedral) data, the results of this function, will be rounded up to the next power of two. The resulting resolution is visualized in figure S3. There, we can see that as the sample size grows, the resolution is increased stepwise from $r_{min} = 256$ to $r_{max} = 4096$. As a result, the effective resolution between increases from $\mathrm{r}_{eff,min} \simeq 85$ to $\mathrm{r}_{eff,max} \simeq 1365$. These numbers translate to an equivalent bin width of at least $\sim 4°$ down to $\sim 0.25°$. We believe this resolution is quite reasonable, given the possible spread of dihedral distributions. This empirical equation has

been proven to lead to very robust results for data sets of basically arbitrary size, as shown in figure S4. Lastly, it is still up to the user to decide against our automatic determination of the resolution and to use a higher or lower resolution. The user is also allowed to go beyond the maximum we chose. Furthermore, we want to note that in both cases, for arbitrary data and for dihedral data, we use these functions as we have found them to be a good compromise between accuracy and speed. That we heavily used numbers that have two as a base is due to the Fast Fourier Transform, which is optimized towards these numbers.

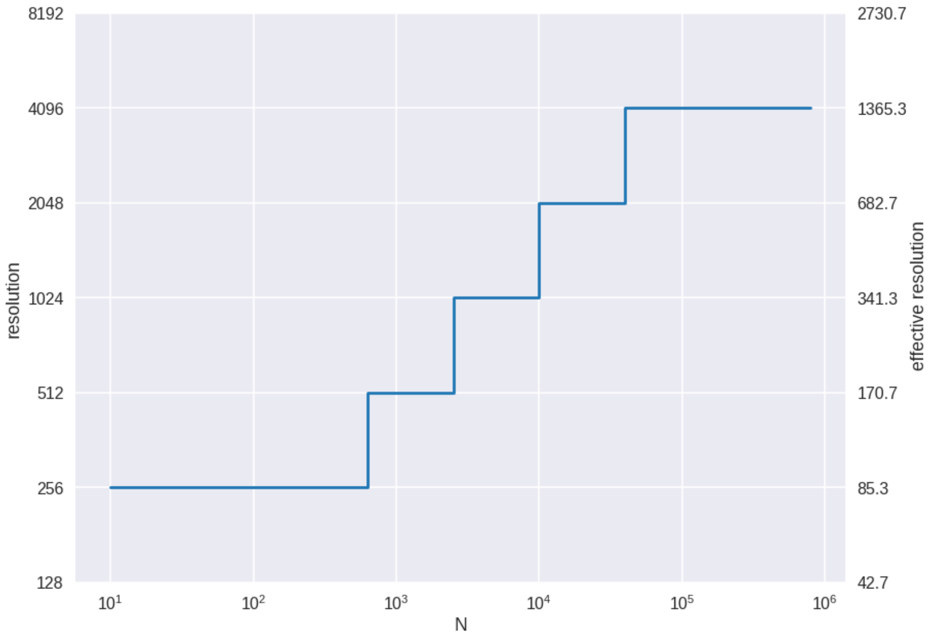

Figure S3: Resolutions obtained for different sample sizes using the implemented rule of thumb, as mentioned in the text. For dihedrals, the resolution states the resolution over the entire binned axes, whereas the effective resolution is only the resolution within the 360° that are used for the calculation.

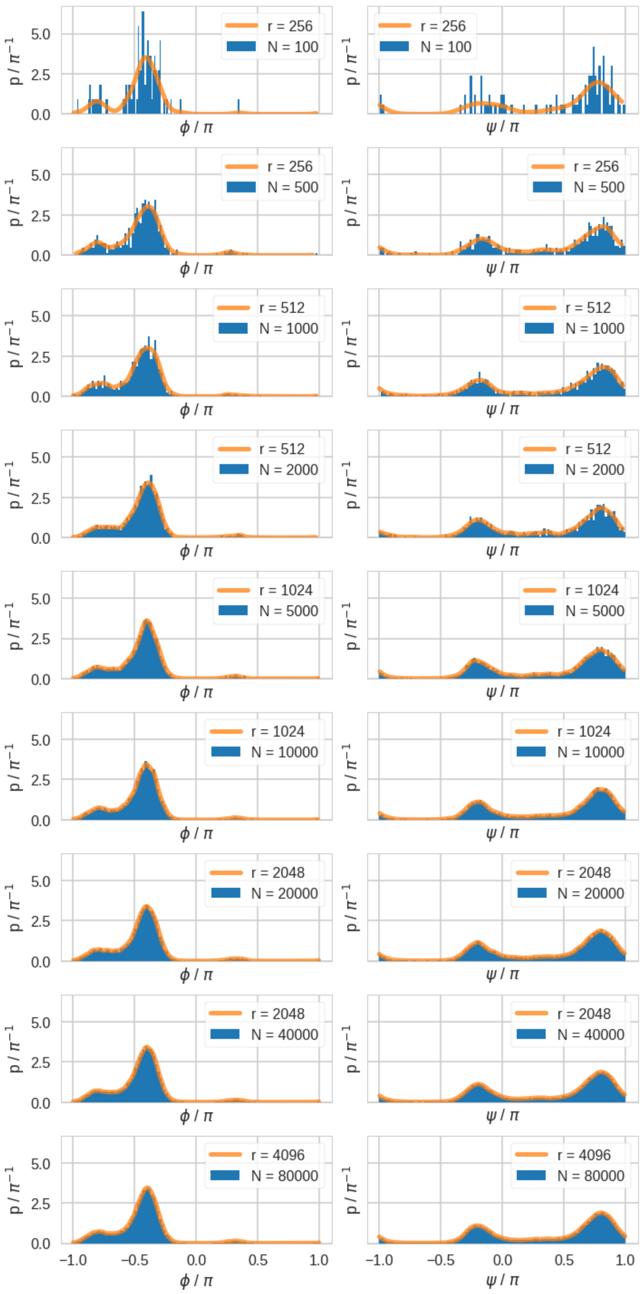

Figure S4: Performance of **X-Entropy** for various sample sizes and resolutions, we compare the underlying data with the KDE for various sample sizes. Ideally, the sample size would have no impact on the KDE, meaning that smaller sample sizes do not decrease the accuracy of the KDE. On the left, we always depict ϕ and on the right side we always depict ψ. The underlying data was plotted as histogram in blue. The calculated KDE is shown as an orange line in front of the underlying data. The sample sizes grow from top to bottom, as does the resolution. The resolution was calculated from the data as mentioned above.

7 KDE Modules

We chose a set of different state-of-the-art modules that can calculate a KDE given any input data. The selection of these was motivated by the fact that all of these packages are considered somewhat a standard to any Python library. In the following, we quickly describe the different methods of the packages to arrive at the optimal bandwidth for a given distribution.

Scipy provides the possibility to either specify the bandwidth by a scalar or to let it be estimated by different rules of thumb. Furthermore, a set of values can be given on which the PDF will furthermore be evaluated. By that, the resolution of the resulting PDF is implicitly given with the latter input.

Sklearn needs a specification of the bandwidth by the user. Similar to scipy, the user also needs to give a set of points for which the logarithm of the probability density will be returned. From these results the PDF can easily be calculated. Also here, the resulting resolution is given by the user's input for the latter routine.

X-Entropy calculates the bandwidth with plug-in selection (see above). Apart from the sampled data, the user may either give a resolution explicitly, or let it be estimated by a rule of thumb (see above). The default procedure to estimate the resolution for arbitrary data has been proven to be quite robust.

8 Alanine Dipeptide simulations

The starting structure for the alanine dipeptide simulation was prepared with the LEaP program of AmberTools 20.[7] The ff14SB force field was used to describe the peptide.[8] The peptide was places in a cubic TIP3P water box with a minimum wall distance of 20 Å.[9] The system underwent an elaborate equlibration protocol prior to production simulation.[10]

Simulations were performed with the GPU implementation of the pmemd module of AMBER 20.[7] A Langevin thermostat was used to keep a constant temperature of 300 K, using a collision frequency of 5 ps^{-1}.[11] Constant atomospheric pressure was maintained with a Monte Carlo barostat. All bond lengths involving hydrogens were contstrained with the SHAKE algorithm[12] to allow the use of a 2 fs timestep. Long range electrostatics were treated with the particle-mesh Ewald approach[13] and a non bonded cutoff of 8 Å was used. All simulations were run for a total length of 2 µs, collecting frames every 10 ps.

Furthermore, we performed accelerated MD (aMD) simulations of 2 µs length, using the dihedral boost option in the AMBER 20 implementation.[14] Boosting parameters were obtained following the protocol suggested by Pierce et al..[15]

8.1 Probability Density Distributions

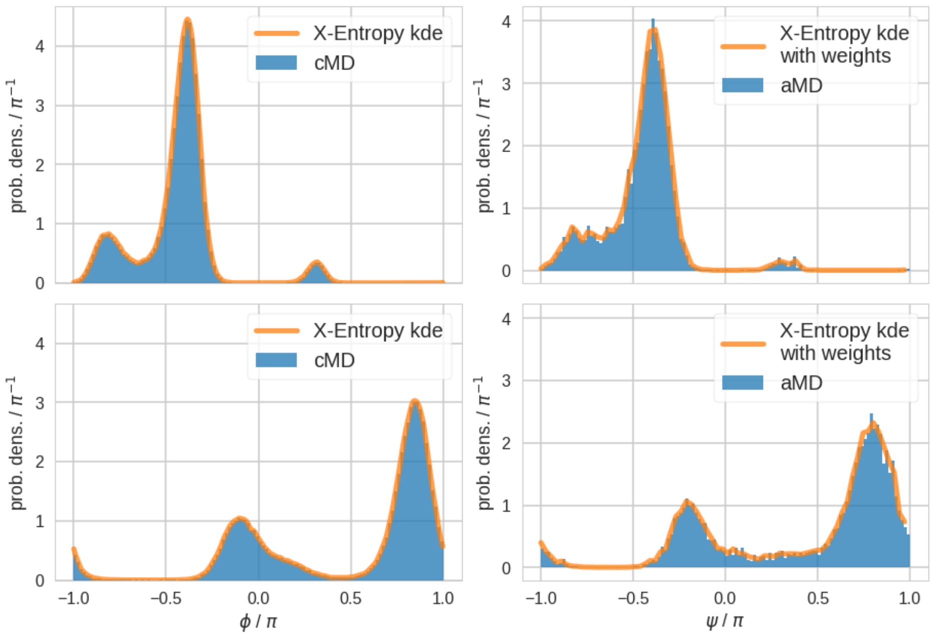

Figure S5: Probability densities of distributions of the dihedral angles of alanine dipeptide, ϕ (top) and ψ (bottom). In blue bars we show the normalized histogram of the data. In orange we show the estimated PDF, which we obtained with **X-Entropy**. In the left panels we show the distribution from cMD simulations, in the right panels, from aMD simulations, respectively.

8.2 Ramachandran plot

We show the Ramachandran plot of the simulations of Alanine Dipeptide in figure S6 and figure S7, respectively.

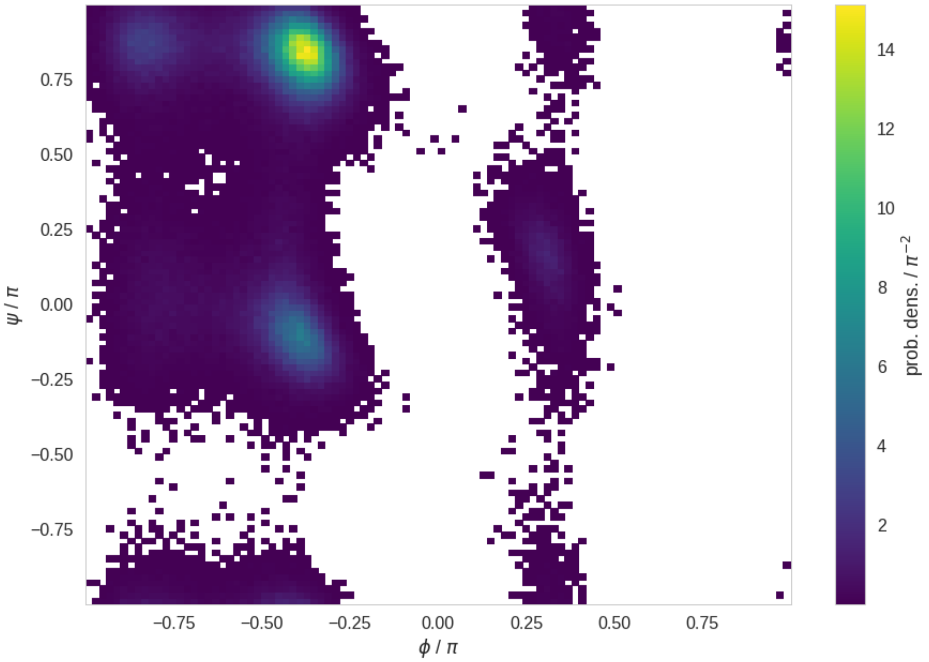

Figure S6: Ramachandran plot for the Alanine Dipeptide cMD simulation.

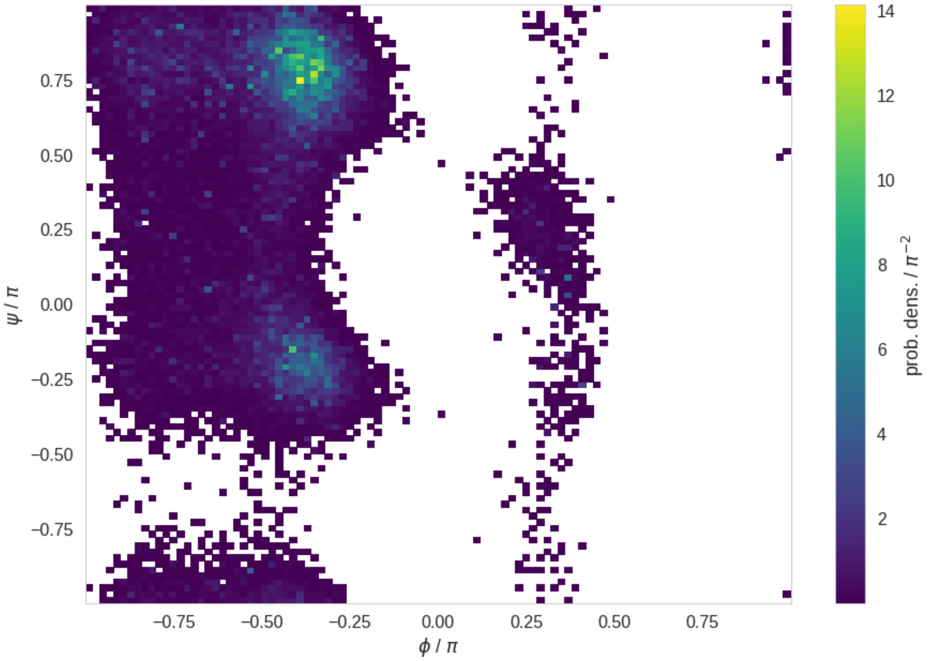

Figure S7: Ramachandran plot for the Alanine Dipeptide aMD simulation.

8.3 Dihedral Entropies

The processing of the data of the 2 μs cMD simulation took $\sim$ 2.5 s altogether (200,000 data points for each dihedral). With this resolution ($r = 4096$), we obtained dihedral entropies of $S_\phi = 4.81$ J/(mol·K) and $S_\psi = 8.22$ J/(mol·K), respectively. This is very close to the values found with aMD, namely 5.09 and 9.62, respectively. It seems that in this case the entropy in the ϕ angles is faster converged than the value for the ψ angle.

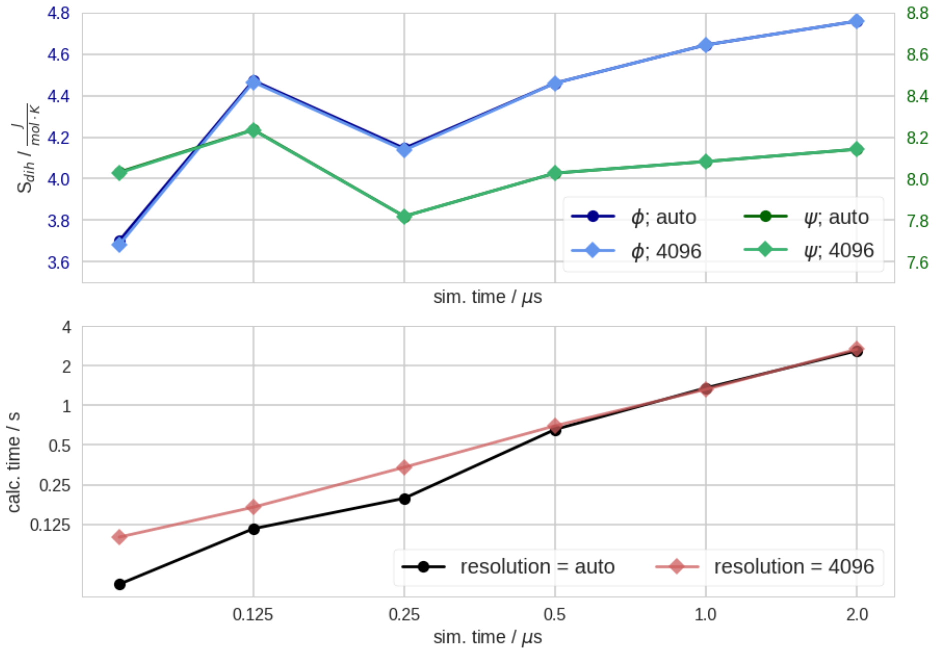

Figure S8: Entropy calculation of cMD simulation of alanine dipeptide for the dihedrals ϕ and ψ. In the upper panel we show the result of the entropies with increasing simulation time: In blue we show ϕ and in green ψ. We use dark colors for results obtained with automatic resolution; in light colors with fixed resolution, r=4096. In the lower panel we show the calculation time for **X-Entropy**.

9 BPTI

The BPTI simulation from the D.E. Shaw Research laboratory was used to analyze a large cMD simulation, where the free energy surface is known. The trajectory consists of 103105 frames, making up $\approx 1\,\mathrm{ms}$.

Figure S9 shows the entropy and the calculation time as a function of the simulated time. We compare these results, when using our automated method to calculate the resolution and setting it directly to 4096. The automatically calculated resolution is very well in line with the results for the higher resolution, however the difference in calculation time is quite substantial. Calculation of the total entropy for the entire system, meaning a summation over all considered backbone dihedrals, yields an entropy value of $74.7\,\frac{\mathrm{J}}{\mathrm{mol\,K}}$. We report a wall clock time of 75.6 s, for the 103105 frames.

Additionally, we compared the dihedral entropy metric from this module to the RMSD as another structural descriptor. We calcualted the RMSD of the atomic displacement between two structures. To compare the two, we used a running window average scheme. For the RMSD, we simply averaged the values inside that window. For the dihedral entropy, we used the trajectory snapshots within this window for the calculation of the entropy. The calculation of the window average models different conformational states. This allows us to analyze the different states in dihedral entropy and RMSD. To calculate the dihedral angles, we used the cpptraj program. We subsequently analyzed them using this module in Python.

Figure S10 shows nicely that in many cases, a large difference in the RMSD is also accompanied by a large difference in the dihedral entropy. Although, at first glance this seems somewhat intuitive, a correlation between the dihedral entropy and the RMSD cannot be expected. The dihedral entropy measures the rotation of the dihedral angles in the backbone of the protein, the RMSD is a less local quantity and measures the mean difference between the structures. So, while two different clusters might be very different in their RMSD, both of them could have a similar dihedral entropy, if they are similar in their flexibility. Figure S11 shows the correlation between the two values, using a linear regression. This figure

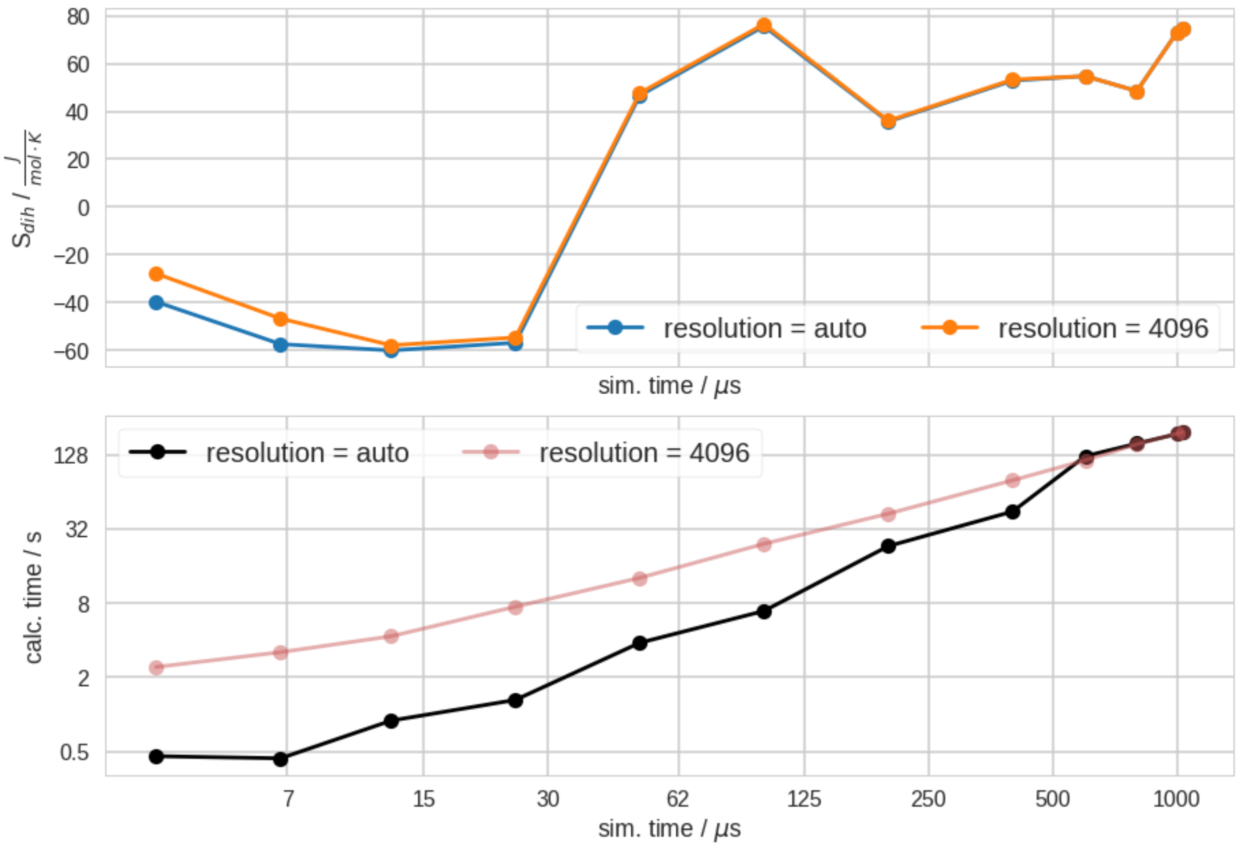

Figure S9: Entropy calculation of the cMD simulation of BPTI for backbone dihedrals ϕ and ψ. The upper panel shows the resulting entropies with increasing simulation time: In blue we show results obtained with automatic resolution; in orange with fixed resolution, r=4096. The lower panel shows the calculation time for **X-Entropy**.

shows the same pattern as figure S10. The R^2 for the dihedral entropy plotted against the RMSD values, is 0.72. On first glance, this is not that large a correlation, but considering the points made before, this is actually a surprisingly high correlation.

To analyze the entropy of different conformational states in BPTI, we compare the dihedral entropy over different parts of the trajectory and the RMSD to the crystal structure in the same parts. Interestingly, we find a high correlation between the two, where structures that are similar to the crystal structure seem to be more rigid than structures that are dissimilar. Generally, it is interesting to observe a strong correlation bewtween the RMSD and dihedral entropy. However, we want to note that this is not necessarily the case, as the dihedral entropy depends solely on the flexibility of the underlying dihedral. This analysis

shows that we are indeed able to characterize different conformational states with respect to their dihedral entropy.

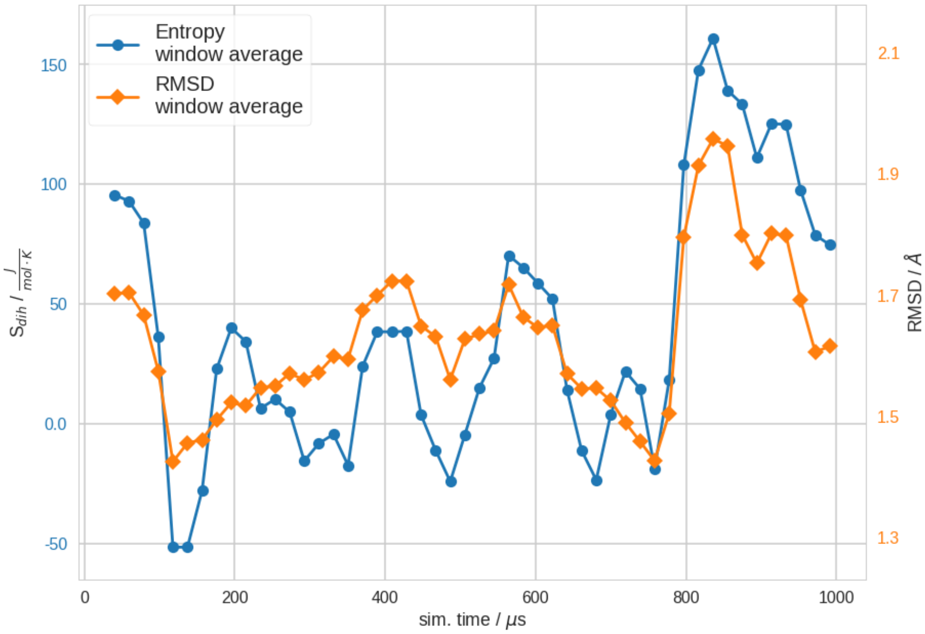

Figure S10: Entropy and RMSD calculation for the BPTI simulation. We show the calculated RMSD (orange) compared to the entropy (blue) averaged over different windows in the trajectory. We used the automatic resolution for the entropy calculation.

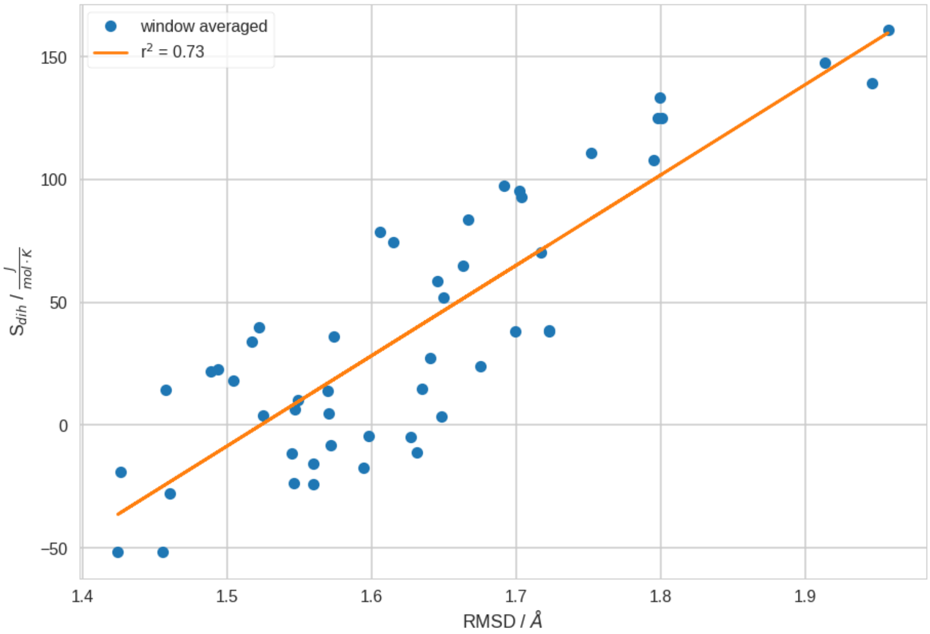

Figure S11: Correlation of entropy and RMSD calculation. We plotted the dihedral entropy against the RMSD values (blue dots). We analyzed the correlation between the two metrics by calculating the r^2. We report a decent correlation of 0.73 for these metrices.

References

(1) Gyimesi, G.; Závodszky, P.; Szilágyi, A. Calculation of Configurational Entropy Differences from Conformational Ensembles Using Gaussian Mixtures. *Journal of Chemical Theory and Computation* **2017**, *13*, 29–41, PMID: 27958758.

(2) Wang, J.; Brüschweiler, R. 2D Entropy of Discrete Molecular Ensembles. *Journal of Chemical Theory and Computation* **2006**, *2*, 18–24, PMID: 26626374.

(3) Botev, Z. I.; Grotowski, J. F.; Kroese, D. P. Kernel density estimation via diffusion. *Ann. Statist.* **2010**, *38*, 2916–2957.

(4) Harris, C. R. et al. Array programming with NumPy. *Nature* **2020**, *585*, 357–362.

(5) Wand, M. P. Data-Based Choice of Histogram Bin Width. *The American Statistician* **1997**, *51*, 59–64.

(6) Freedman, D.; Diaconis, P. On the histogram as a density estimator: L2 theory. *Zeitschrift fuer Wahrscheinlichkeitstheorie und Verwandte Gebiete* **1981**, *57*, 453–476.

(7) Case, D. et al. AMBER 2020. 2020.

(8) Maier, J. A.; Martinez, C.; Kasavajhala, K.; Wickstrom, L.; Hauser, K. E.; Simmerling, C. ff14SB: Improving the Accuracy of Protein Side Chain and Backbone Parameters from ff99SB. *Journal of Chemical Theory and Computation* **2015**, *11*, 3696–3713.

(9) Jorgensen, W. L.; Chandrasekhar, J.; Madura, J. D.; Impey, R. W.; Klein, M. L. Comparison of simple potential functions for simulating liquid water. *The Journal of Chemical Physics* **1983**, *79*, 926–935.

(10) Wallnoefer, H. G.; Handschuh, S.; Liedl, K. R.; Fox, T. Stabilizing of a Globular Protein by a Highly Complex Water Network: A Molecular Dynamics Simulation Study on Factor Xa. *The Journal of Physical Chemistry B* **2010**, *114*, 7405–7412.

(11) Adelman, S. A.; Doll, J. D. Generalized Langevin equation approach for atom/solid-surface scattering: General formulation for classical scattering off harmonic solids. *The Journal of Chemical Physics* **1976**, *64*, 2375–2388.

(12) Ryckaert, J.-P.; Ciccotti, G.; Berendsen, H. J. Numerical integration of the cartesian equations of motion of a system with constraints: molecular dynamics of n-alkanes. *Journal of Computational Physics* **1977**, *23*, 327–341.

(13) Darden, T.; York, D.; Pedersen, L. Particle mesh Ewald: An Nâ‹. . . log(N) method for Ewald sums in large systems. *The Journal of Chemical Physics* **1993**, *98*, 10089–10092.

(14) Hamelberg, D.; Mongan, J.; McCammon, J. A. Accelerated molecular dynamics: A promising and efficient simulation method for biomolecules. *The Journal of Chemical Physics* **2004**, *120*, 11919–11929.

(15) Pierce, L. C.; Salomon-Ferrer, R.; Augusto F. de Oliveira, C.; McCammon, J. A.; Walker, R. C. Routine Access to Millisecond Time Scale Events with Accelerated Molecular Dynamics. *Journal of Chemical Theory and Computation* **2012**, *8*, 2997–3002.
